# Taxonomic Insights and Its Type Cyclization Correlation of Volatile Sesquiterpenes in *Vitex* Species and Potential Source Insecticidal Compounds: A Review

**DOI:** 10.3390/molecules26216405

**Published:** 2021-10-23

**Authors:** Ighor C. Barreto, Anderson S. de Almeida, José G. Sena Filho

**Affiliations:** 1 Coordenação de Meio Ambiente, Universidade Federal da Bahia, Av. Adhemar de Barros, Ondina, Salvador 40170-110, BA, Brazil; 2 Programa de Pós-Graduação em Ciências Farmacêuticas, Universidade Federal de Sergipe, Av. Marechal Rondon, Rosa Elze, São Cristóvão 49100-000, SE, Brazil; anderson123soares@outlook.com; 3 Empresa Brasileira de Pesquisa Agropecuária-EMBRAPA Coastal Tablelands, Av. Beira mar, 3250, Aracaju 49025-040, SE, Brazil; jose-guedes.sena@embrapa.br

**Keywords:** *Vitex*, biosynthesis, sesquiterpenes synthases, cyclization

## Abstract

Sesquiterpenes (SS) are secondary metabolites formed by the bonding of 3 isoprene (C5) units. They play an important role in the defense and signaling of plants to adapt to the environment, face stress, and communicate with the outside world, and their evolutionary history is closely related to their physiological functions. This review considers their presence and extensively summarizes the 156 sesquiterpenes identified in *Vitex* *taxa*, emphasizing those with higher concentrations and frequency among species and correlating with the insecticidal activities and defensive responses reported in the literature. In addition, we classify the SS based on their chemical structures and addresses cyclization in biosynthetic origin. Most relevant sesquiterpenes of the *Vitex* genus are derived from the germacredienyl cation mainly via bicyclogermacrene and germacrene C, giving rise to aromadrendanes, a skeleton with the highest number of representative compounds in this genus, and 6,9-guaiadiene, respectively, indicating the production of 1.10-cyclizing sesquiterpene synthases. These enzymes can play an important role in the chemosystematics of the genus from their corresponding routes and cyclizations, constituting a new approach to chemotaxonomy. In conclusion, this review is a compilation of detailed information on the profile of sesquiterpene in the *Vitex* genus and, thus, points to new unexplored horizons for future research.

## 1. Introduction

Volatile sesquiterpenes, like all terpenoids, are derived from the five-carbon precursor isopentenyl diphosphate (IPP) and its isomer dimethylallyl diphosphate (DMAPP) [1,2]. Plant species use two separate pathways to synthesize these precursors: the mevalonate acid pathway (MVA), which is located in the cytosol and partially in the endoplasmic reticulum and peroxisomes, and the methylerythritol phosphate pathway (MEP), which is located in the plastids [2,3,4].

For the biosynthesis of volatile sesquiterpenes, farnesyl diphosphate synthase (FDS), a branch point enzyme in the biosynthesis of these terpenoids, condenses a DMAPP unit with two IPP units to form the linear precursor farnesyl diphosphate (*E*,*E*-FPP, C15). This, by cleavage, forms a reactive carbocation, which undergoes electrophilic cyclization and rearrangements to form sesquiterpenes (SS) through a cascade of enzymatic reactions catalyzed by families of functionally distinct enzymes of sesquiterpene synthase (sesqui (TPS)) and cytochrome P450 mono-oxygenase (P450), which are the main drivers of skeletal formation and functional modifications, respectively [5,6,7]. The cascade of reactions generated by sesqui (TPS) proceeds through the intermediate carbocations, which serve as ramifications for specific pathways in the chemical cascade [1,8]. In general, the proposed reaction mechanism for SS formation consists of three main stages: (1) generation of a carbocation, (2) hydride changes and carbocation rearrangements, and (3) neutralization of a carbocation by deprotonation or capture of a nucleophile (e.g., water) [9,10].

**Figure 1:**
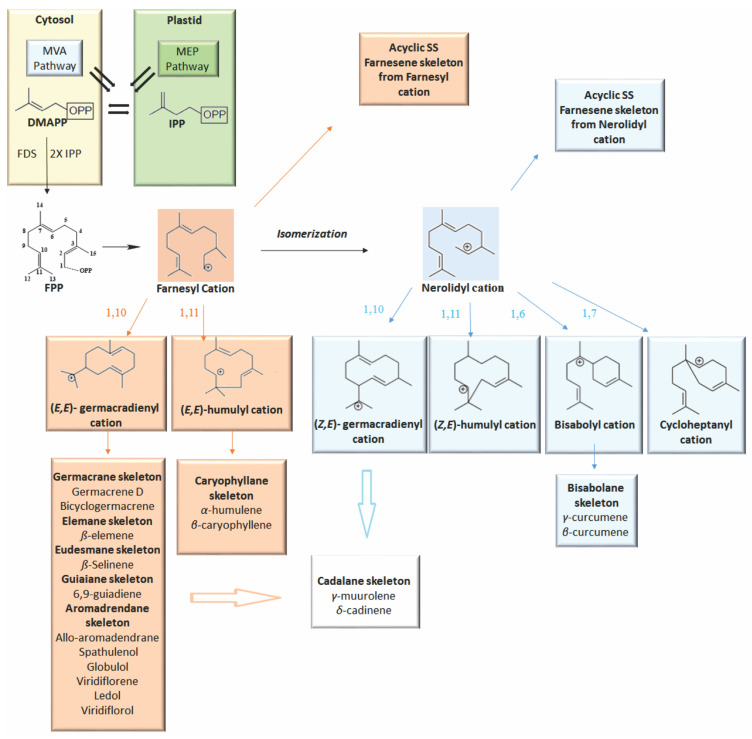
Biosynthesis of sesquiterpenes in *Vitex* species.

Alternatively, sesqui (TPS) can use a secondary carbocation formed from the isomer (*E*,*E*)-FPP, the (3*R*)-nerolidyl diphosphate (3*R*-NPP), and then proceed to the formation of the terpenoid skeleton. The first cyclization that occurs by attacking the double bond with carbocations derived from (*E*,*E*)-FPP or (3*R*)-NPP (farnesyl or nerolidyl cation) can be used to divide the sesquiterpenes produced by plants into seven groups, which can be 1.10 or 1.11 of the farnesyl carbocation or 1.6, 1.7, 1.10, 1.11-cyclization of the nerolidyl carbocation (Figure 1) [11,12].

Sesquiterpenes have more than 7000 identified carbon skeletons from different organisms [13]. In plants, volatile SS hydrocarbons are well known as constituents of essential oils and play ecological roles in the plant’s interaction with pollinators and predators. Many of these compounds are released by flowers to attract pollinators [14] and play an important role in direct and indirect chemical defense against herbivores and phytopathogens [15,16,17]. They are the volatile constituents released by plants defense after attack by herbivores, attracting arthropods that attack or parasitize these herbivores [15,18,19,20]. In addition, they are also synthesized and accumulated in organs such as rhizomes and roots, participating in the attraction of nematode predators [17,21].

*Vitex* (Lamiaceae, Viticoideae) comprises c. 250 pantropical, subtropical, and some temperate species [22]. The most common species known for their medicinal properties are *V. agnus-castus*, *V. rotundifolia,* and *V. negundo* [22]. According to our survey, 21 *Vitex* species have essential oils reported in the literature database. These species have a diversity of volatile terpenes, mainly sesquiterpenes, which are present in great abundance [23,24,25,26,27,28,29,30]. This genus also has some nonvolatile sesquiterpenoids. Yao et al. [30] published a review of terpenes obtained from *Vitex* species. They reported that eight SS structures were obtained, including a structure containing a furan ring, three furanoeremophylane, and four sesquiterpenoids with an aromadendrane skeleton with a seven-membered ring. Interestingly, volatile SS varieties with a seven-membered ring aromadendrane skeleton were found in *Vitex* species in different regions of the world [25,30,31,32,33,34]. It was hypothesized that sesqui (TPS) that are being expressed in the genus *Vitex*, which are responsible for the formation of compounds with fused five- and seven-membered rings, may play an important role in chemosystematics [25].

There was great progress in recent years in the identification and functional characterization of genes for the biosynthesis of SS and cyclase enzymes, which led to a greater understanding of the mechanisms and variability of biosynthesis of these terpenoids [7,35,36]. So far, a large number of sesqui (TPS) responsible for the formation of defensive SS were cloned and functionally characterized from various plants, such as corn, rice, sorghum, cotton, and tomatoes [7,37,38,39,40]. Defenses related to SS were well described in these species of angiosperm, revealing several chemical mechanisms for resistance against above and below ground stressors, providing much stronger evidence for the involvement of SS in plant defense [37,41,42,43]. This knowledge can be combined with versatile metabolic engineering approaches for the broader production of terpenoid bioproducts [44]. Although advances have occurred, there is still a vast field of knowledge about the gene structure, catalysis mechanism, and expression regulation for a large number of sesqui (TPS) from various plants, including *Vitex* species.

In this context, this review addresses the possible sesqui (TPS) that are being expressed in the genus *Vitex*, the type of cyclization that occurs in the biosynthetic origin of SS, which were identified with frequency and high concentrations in species, and its correlation with the insecticidal activities and defensive responses reported in literature. This paper covers the literature database correlating sesquiterpenes/sesquiterpenes synthases, *Vitex* species and insecticidal activities. This review is a valuable source of information in the field of plant SS biosynthesis, and therefore we compiled detailed information on the profile of SS in the genus *Vitex* and, thus, also indicated new unexplored horizons for future research.

## 2. Volatile Sesquiterpenes in *Vitex* Genus

Usually, SS are classified based on different oxygen functions, such as alcohol, aldehyde, and sesquiterpene lactone. This is relevant to their physiological activities and physical and chemical properties [45]. They are also classified by the number of carbon rings in their chemical structure, such as acyclic, monocyclic, bicyclic, tricyclic, and tetracyclic [46]. In addition, SS can also be classified according to the number of carbons in the rings, with most rings containing 5, 6, 7, and up to 11 carbons [47].

**Figure 2:**
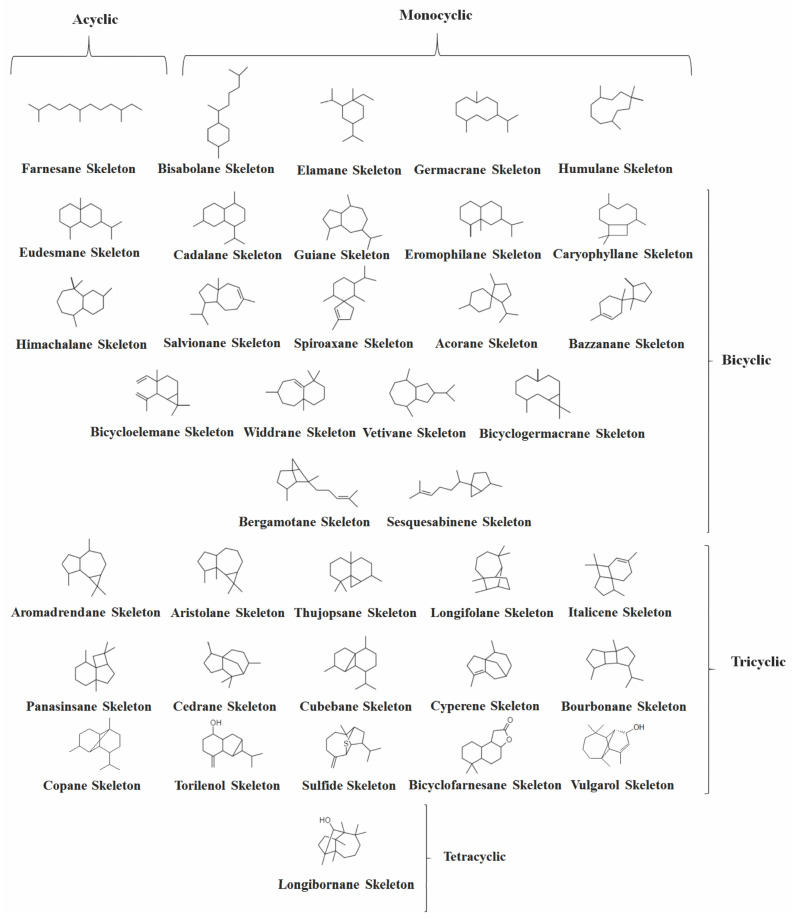
Structures of parent carbon skeletons of all sesquiterpenes identified in *Vitex*.

**Figure 3:**
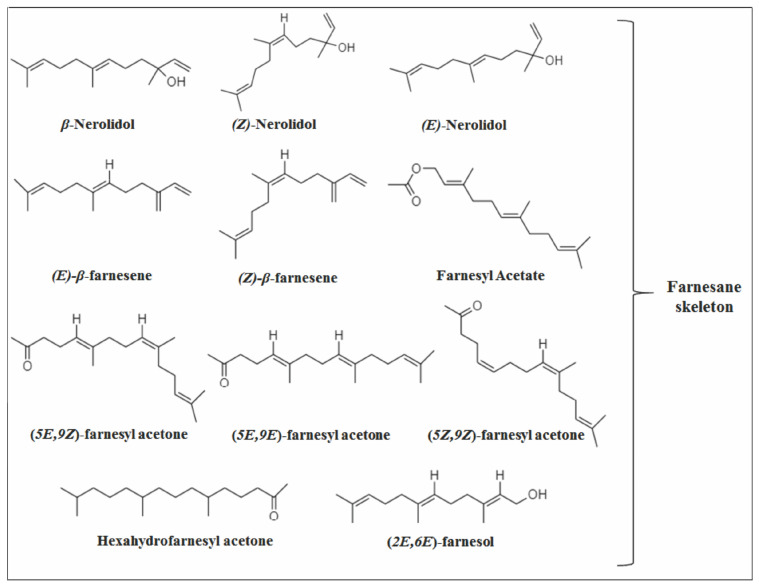
Structure of acyclic sesquiterpenes in *Vitex* species.

**Figure 4:**
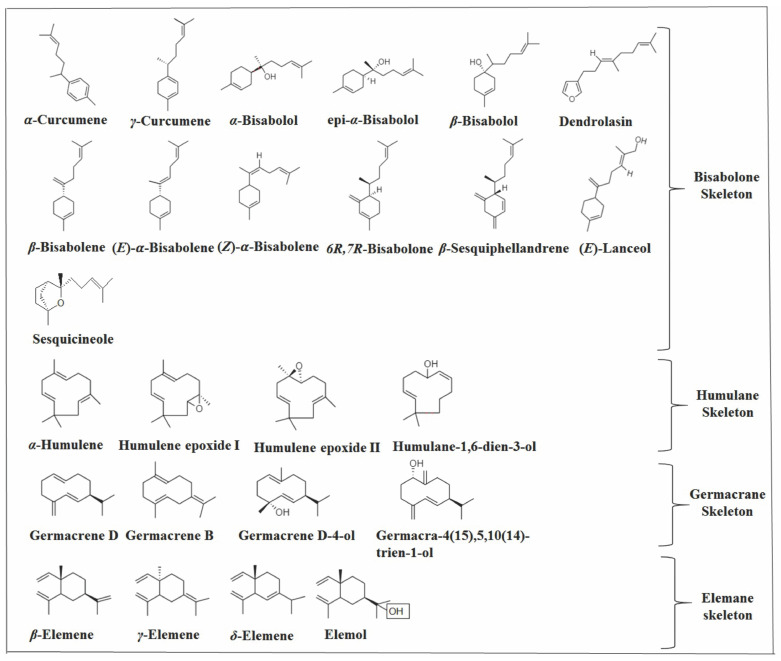
Structures of monocyclic sesquiterpenes in *Vitex* species.

Several investigations were carried out on the chemical composition of different *Vitex* species from different geographic regions. As far as we know, 156 volatile SS were identified in *Vitex* species (Figures 3, 4, 9 and 13), which are distributed in 37 skeletons (Figure 2). Among them, the bicyclic SS cadalane type is the one with the highest number of compounds identified in the *Vitex* genus followed by the eudesmane and the tricyclic aromadrendane. However, bicyclic caryophyllane-type compounds, such as (*E*)-*β*-caryophyllene (EβC), caryophyllene oxide, and the monocyclic *α*-humulene, were the most representative volatile SS within the *Vitex* genus, appearing in many species in high concentrations.

Next, the SS of the *Vitex* species were classified based on the number of carbon rings and subclassified by the original carbon skeletons on which their chemical structures are based according to the work of [47], highlighting those that appeared more often and in high concentrations. Furthermore, the type of primary cyclization in the biosynthetic origin of these compounds was suggested.

### 2.1. Acyclic Sesquiterpenes

The acyclic group has the smallest number of members, with only 11 acyclic SS identified in *Vitex* species, and all containing a farnesane skeleton (Figure 3). Among them, the compound (*E*)-*β*-farnesene (EβF) stands out, which is reported in eight species, being one of the main components of *V. agnus-castus* in various regions of the globe [24,26,31,48,49,50,51,52,53,54,55,56,57,58,59,60]. Probably, EβF synthase is being expressed in this species.

The gene-encoding EβF synthase, which catalyzes the formation of EβF, was identified and characterized for the first time from *Mentha piperita* L. [61]. Later, orthologous EβF synthase genes were isolated from other plants, such as *Citrus junos* [62], *Pseudotsuga menziesii* [63], *Matricaria recutita* [64], and *Artemisia annua* [65,66].

The acyclic pathway begins with the addition of water or the loss of protons from the carbocation farnesyl or nerolidyl [12,36]. In this pathway, the carbocation does not undergo a cyclization process as in other pathways, being responsible for the production of several acyclic SS from the farnesane skeleton [47].

### 2.2. Monocyclic Sesquiterpenes

There are 24 monocyclic sesquiterpenes that were identified in *Vitex* species. They can be classified into four subcategories based on the carbon skeleton, such as humulane, germacrane, elemane, and bisabolane (Figure 4).

#### 2.2.1. Humulane Skeleton

Four compounds with a humulane skeleton were identified (Figure 4). Among them, *α*-humulene, which was reported in 17 *Vitex* species, is one of the main compound in *V capitata*, *V. megapotamica*, *V. rufecens* [25,67], *V. simplicifolia* [68], and *V. doniana* [28].

Although *α*-humulene is a common SS in plants, only *α*-humulene synthase was identified in the species *Zingiber zerumbet*, *Picea glauca*, and *Aquilaria crassna*, catalyzing the formation of *α*-humulene as the main product and *β*-caryophyllene as the secondary product [69,70]. However, in *Vitex* species, *α*-humulene was identified as a secondary product or in smaller amounts and EβC was identified as the main compound, while *α*-copaene and *β*-elemene were also identified in smaller amounts. Interestingly, sequi (TPS) capable of producing these compounds in this way was described and identified in plant species *Arabidopsis thaliana* (AtTPS21) and *Oryza sativa* (OsTPS3) as (*E*)-*β*-caryophyllene synthase (EβCs) [16,71]. Other studies reported that this synthase catalyzed the formation of EβC as a major product and *α*-humulene in smaller amounts [72,73,74].

**Figure 5:**
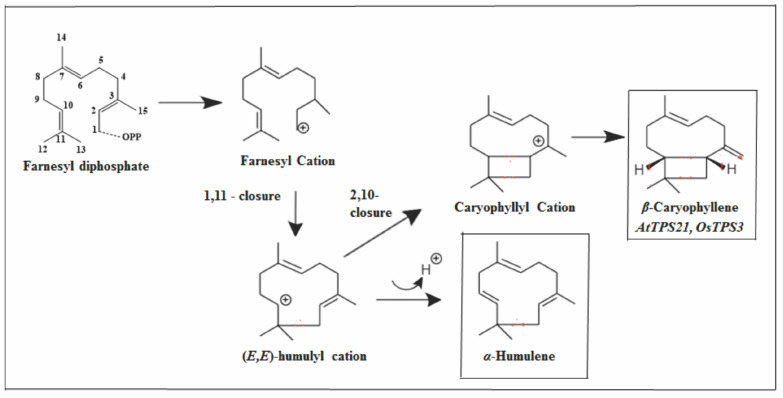
Types of primary cyclization of *α*-humulene and *β*-caryophyllene.

The origin of these SS is the result of 1.11-cyclization to form a humulyl cation, which by deprotonation of C-9 can form *α*-humulene or promote the closure of 2.10 generating EβC (Figure 5) [69,75].

#### 2.2.2. Germacrane Skeleton

Germacrenes are a subclass of SS with a germacrane skeleton. Four compounds with this skeleton were identified in *Vitex* species (Figure 4). However, germacrene D is the most relevant compound, appearing in eleven species, and is the major compound in the essential oils of *V. rivularis* and *V. ferruginea* [29,30], with significant amounts in *V. rufescens* and *V. simplicifolia* [25,68]. Due to the high concentration of this SS, germacrene D synthase is possibly being expressed in *V. rivularis* and *V. ferruginea*. The gene (FcTPS1) encoding this synthase in *Ficus carica* L. catalyzed the predominant formation of germacrene D together with *α*-cubebene, EβC, *γ*-muurolene, *α*-muurolene, *γ*-cadinene, and *δ*-cadinene in smaller amounts [76], as can be seen in *V. rivularis* and *V. ferruginea*.

**Figure 6:**
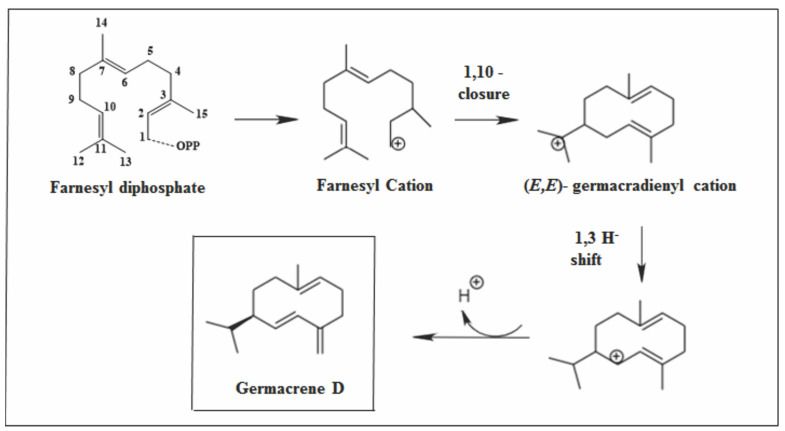
A type of primary cyclization of germacrene D.

Germacrene D is a biogenetic precursor of many SS. This pathway is considered one of the most important, being responsible for the biosynthesis of numerous sesquiterpenes. It can also be classified into three subpathways: via cadinenyl cation, via muurolenyl cation, and via amophenyl cation [77]. The formation of this sesquiterpene occurs through 1.10-cyclization of the farnesyl cation. The subsequent reaction pathway was shown to involve different hydrogen displacements to provide germacrene D (Figure 6) [11,78,79,80].

#### 2.2.3. Bisabolane Skeleton

The bisabolane skeleton had the largest number of compounds among the monocyclic sesquiterpenes. Thirteen compounds were identified in *Vitex* plants (Figure 3). Although the compounds in this group did not show a relevant concentration and frequency among the species, *γ*-curcumene and *β*-curcumene were the secondary and tertiary products of *V. rivularis* [29], respectively. As mentioned earlier, germacrene D is the major compound in this species.

So far, only *γ*-curcumene synthase (PatTpsA) from *Pogostemon cablin* was identified in plants, generating *γ*-curcumene as the only product [81]. Studies by targeting amino acid residues mutation in the active site of the epi-isozyzaene synthase (EIZS) of *Streptomyces coelicolor* converted this enzyme into new sesqui (TPS), including *β*-curcumene synthase (F95H EIZS) and F95Q EIZS (unidentified synthase), generating *β*-curcumene as the main product and the *β* and *γ*-curcumene regioisomers as the main cyclization products, respectively [82,83].

**Figure 7:**
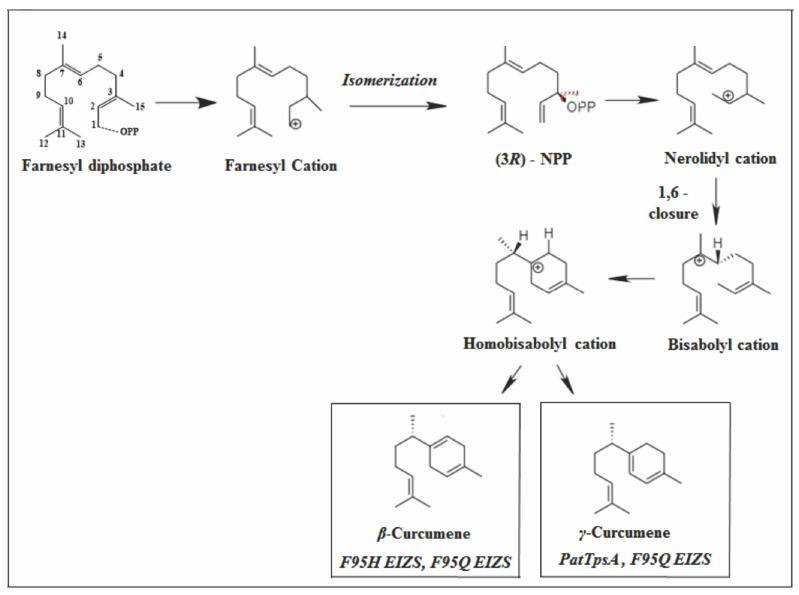
Types of primary cyclization of *γ*-curcumene and *β*-curcumene.

The proposed mechanism for cyclization of curcumene sesquiterpenes derives from 1.6-cyclization to form the bisabolyl carbocation. The displacement of [1,2]-hydride forms the homobisabolyl cation which, due to the loss of the proton, forms the derivatives of curcumene (Figure 7) [82,83,84].

#### 2.2.4. Elemane Skeleton

Four elemane skeletons type compounds were identified in *Vitex* plants (Figure 3). However, only *β* and *γ*-elemene have attracted attention. The first was identified in 10 species, appearing in significant concentrations in *V. quinate* and *V. rufecens* [25,85] and in smaller amounts in *V. capitata* and *V. megapotamica* [25,67]. Its isomer, *γ*-elemene, appears as one of the main compounds in *V. capitata* and in *V. megapotamica* [25,67]. Interestingly, *δ*-elemene appeared as one of the major compounds of *V. megapotamica* collected in southern Brazil [67].

**Figure 8:**
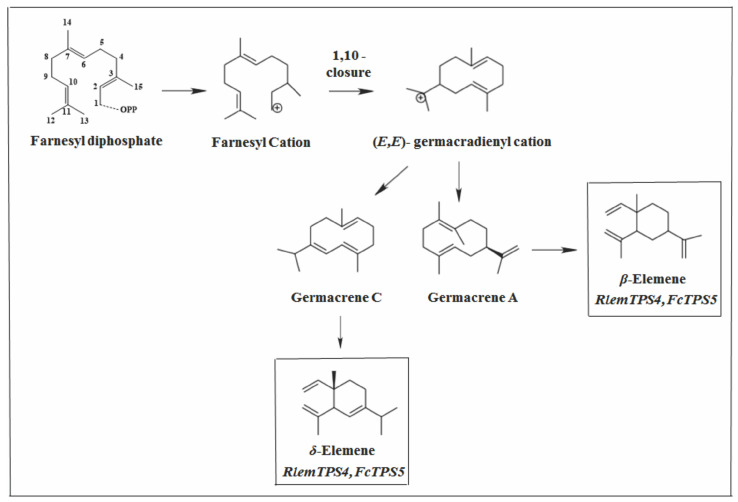
Types of primary cyclization of compound *β*-elemene and *δ*-elemene.

The sesqui (TPS) for *β*-elemene, whose compound is predominant in plants, was identified only in rice [86]. However, *β*-elemene is generally considered a transformation product from germacrene A, which is synthesized by germacrene A synthase (Figure 8) [21,87,88,89].

From a biogenetic point of view, many elemene-type sesquiterpenes are produced from the corresponding germacrenes via Cope rearrangement [90]. Studies showed that during isolation and analysis by gas chromatography (GC), germacrene A undergoes a Cope to *β*-elemene rearrangement induced by heating in the injector [91,92,93,94], while germacrene B and germacrene C rearranges to *γ*-elemene [95] and *δ*-elemene [90], respectively. However, germacrene A was not detected in any of the *Vitex* species. Instead, *β*-elemene appeared as one of the secondary products. This compound probably comes from a single enzyme that uses a single substrate, giving rise to several products [7]. The multiple products are mainly due to the stochastic nature of the linked rearrangements, which follow the creation of the unusual carbocation intermediates before the reaction is terminated through deprotonation or nucleophile capture [7]. As mentioned earlier, EβCs are possibly being expressed in *V. rufescens*, *V. capitata*, *V. megapotamica*, and *V. quinata*. This enzyme catalyzed several products in smaller amounts in other plants, including *β*-elemene [16,71]. On the other hand, the significant concentration of *γ*-elemene and corresponding decrease in its precursor germacrane B in *V. capitata* and *V. megapotamica* [25] may be due to the high temperature of the injector port in the analysis of GC.

The *δ*-elemene that appeared as one of the main products of *V. megapotamica* collected in southern Brazil [67] is probably due to the expression of the gene encoding an *δ*-elemene synthase, emitting the *δ*-elemene as the main compound and *β*-elemene in smaller amounts. Uji et al. [96] was the first to identify a sesqui (TPS) (RlemTPS4) in plants, producing *δ*-elemene as a major product and *β*-elemene as a minor product. Recently, *δ*-elemene synthase (FcTPS5) from *Ficus carica* was identified, which also catalyzed the formation of *δ* and *β*-elemene as main products [76].

### 2.3. Bicyclic Sesquiterpenes

**Figure 9:**
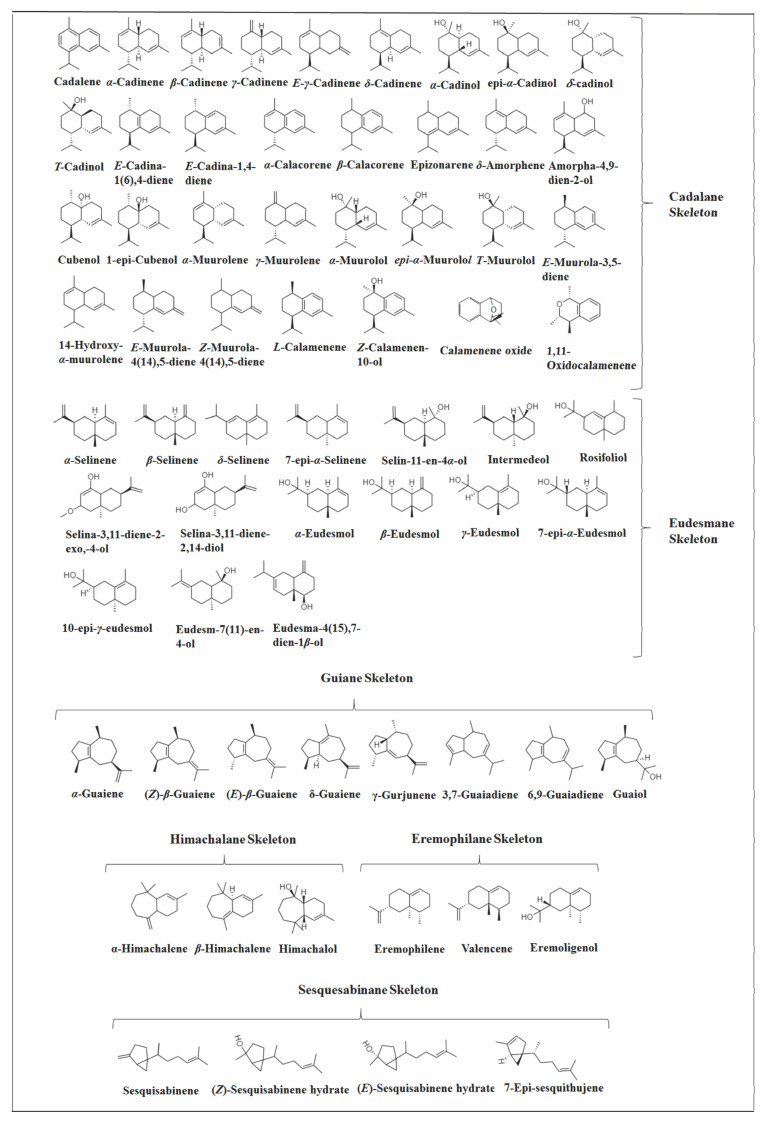

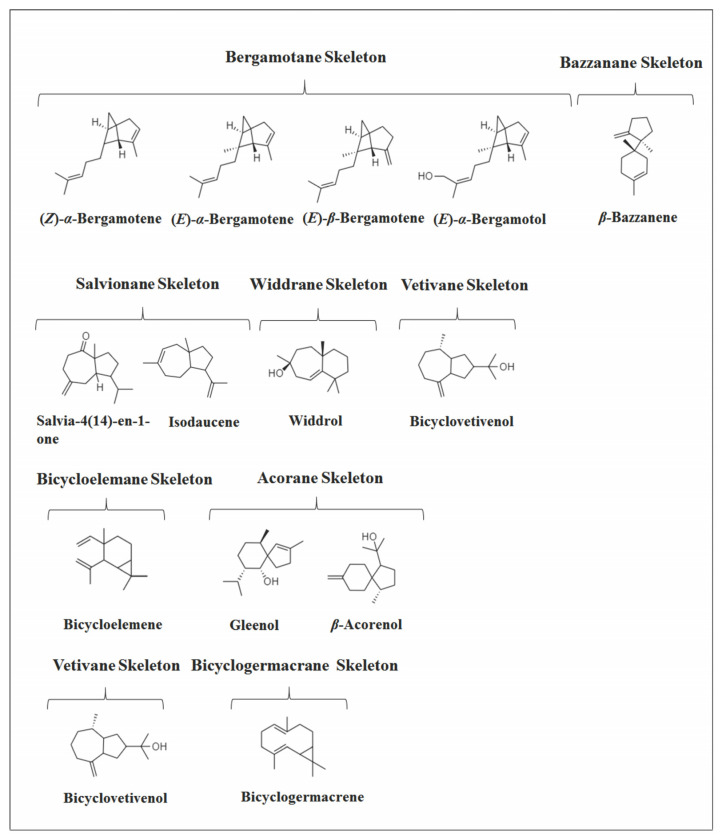
Structures of bicyclic sesquiterpenes in *Vitex* species.

Bicyclic SS represent the largest group in *Vitex* species with 81 identified compounds and can be classified into 11 subcategories based on the carbon skeleton (Figure 9), with eudesmane, caryophyllane, cadalane, and bicyclogermacrene skeletons being the most prevalent.

#### 2.3.1. Cadalane Skeleton

Cadalane skeleton is the group with the highest number of compounds in *Vitex* plants, with 30 structures reported. Following the criterion adopted in this survey of high frequencies and concentrations, the compounds *γ*-muurolene and *δ*-cadinene are the ones that have these characteristics. The first appears in 12 species, while the second was identified in 13 species. Interestingly, both were the main compounds in *V. megapotamica* and *V. capitata* species [25,67]. Other species, such as *V. rivularis*, *V. obovata* ssp. obovata, *V. obovata* ssp. Wilmsii, and *V. ferruginea*, had significant amounts of one of these compounds [29,30,33].

The entire series of cadalanes is generated by the protonation of an intermediate neutral germacrene D [97], which is a potent precursor of cadinenes and muurolenes [95]. Biosynthetic pathways for the formation of *δ*-cadinene and *γ*-muurolene via germacrene D in the legume *Truncatula medicago* were reported [98]. *δ*-cadinene occurs very frequently in plants together with germacrene D when it is in higher concentrations [77]. This can be observed in the species *V. rivularis*, *V. ferruginea*, *V. rufecens*, and *V. simplicifolia* [25,29,30,68]. However, investigations of *δ*-cadinene synthase, which catalyzes the formation of *δ*-cadinene as the main product, as well as a multitude of other sesquiterpenes were reported in the species of laurel (*Leonurus sibiricus*), fig (*Ficus carica*), cotton (*Gossypium hirsutum*), not showing any germacrene D in the products [76,99,100], as well as *V. megapotamica* and *V. capitata* [25,67], which have *δ*-cadinene in larger amounts.

**Figure 10:**
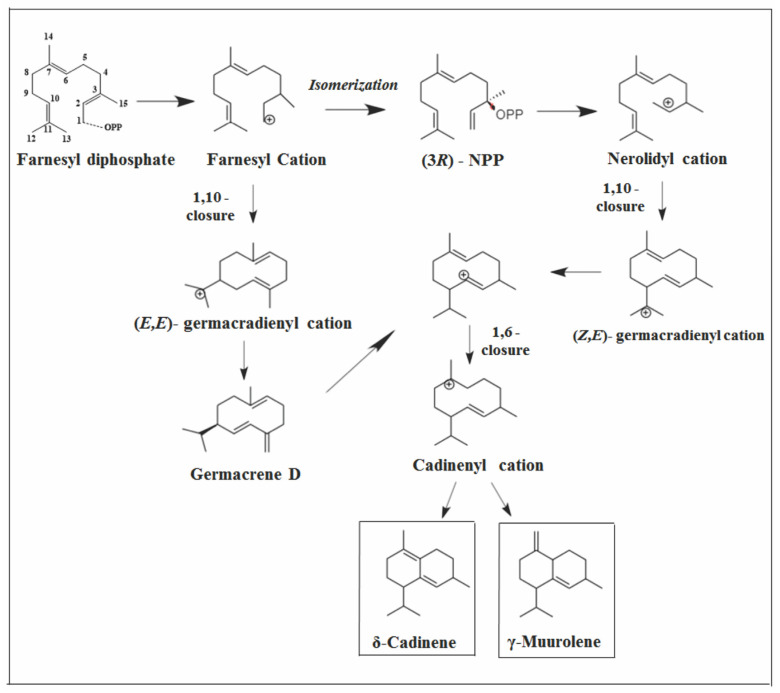
Types of primary cyclization of compounds *δ*-cadinene and *γ*-muurolene.

Cadinene and muurolene skeletons may also result from an earlier rearrangement from farnesyl to the nerolidyl cation [40,101,102,103,104]. Germacradienyl cation forming by 1.10-cyclization. Subsequently, a 1.6-electrophilic ring closure reaction generates the cadinenyl cation from which *δ*-cadinene and *γ*-muurolene are formed (Figure 10) [98].

#### 2.3.2. Caryophyllane Skeleton

SS with a caryophyllane skeleton have 11 compounds identified in *Vitex* species (Figure 4). EβC and caryophyllene oxide are the most relevant in this group. Furthermore, EβC is one of the most representative volatile SS in the *Vitex* genus, appearing in 15 species, and is the major compound in six species: *V. megapotamica*, *V. capitata*, *V. rufescens*, *V. negundo*, *V. trifolia*, and *V. agnus-castus* [25,105,106]. Furthermore, it was identified in high concentrations in *V. quinata* and *V. rivularis* [29,85]. On the other hand, caryophyllene oxide was reported in almost all *Vitex* species except for *V. rotundifolia*. It was one of the main compounds of *V. gardneriana*, *V. negundo*, *V. rehmannii*, *V. obovata* ssp. obovata, *V. pooara*, *V. trifolia*, and *V. kwangsiensis* [25,27,33,106,107].

EβCs were already identified and characterized in several plant species and were extensively reported in the literature [72,73,74,76]. Generally, this enzyme produces EβC as the main product and its *α*-humulene isomer in smaller amounts. EβCs are probably being expressed in *Vitex* species; EβC was identified as the main product and *α* -humulene as the secondary product or in lower concentrations. On the other hand, there are no reports in the literature of specific shyntases for caryophyllene oxide; however, there is a consensus that it is formed by oxidation of EβC [108,109,110].

#### 2.3.3. Eudesmane Skeleton

**Figure 11:**
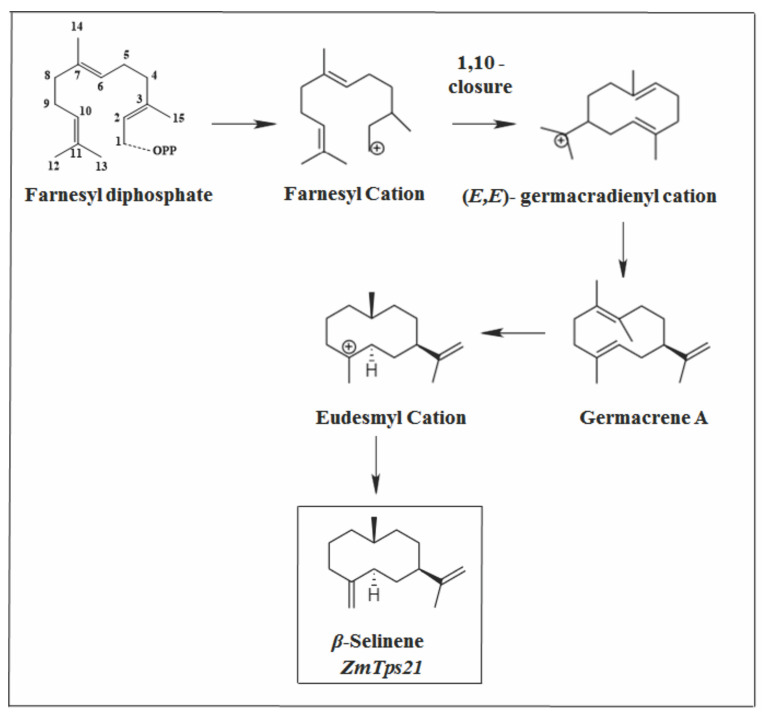
A type of primary cyclization of *β*-selinene.

There are 14 bicyclic sesquiterpenes in *Vitex* species that have the eudesmane parental skeleton (Figure 4). Among them, *β*-selinene appears in 13 species and is the marjority SS in *V. pooara* [33]. ZmTps21 from corn (*Zea mays*) encodes *β*-selinene synthase, producing *β*-selinene as the dominant product along with *β*-elemene at lower concentrations [111]. This can be observed in *V. pooara*, suggesting that this sesqui (TPS) is expressed in this species. *β*-selinene is simply formed by a deprotonation of a eudesmane carbocation, which was reported to originate from germacrene A to form 5-epi-aristolochene [10,111,112]. It is suggested that the primary cyclization that occurs for the formation of *β*-selinene is of type 1.10 (Figure 11).

### 2.4. Other Bicyclic Sesquiterpenes

Bicyclogermacrene is structurally similar to germacrene with a classic bicyclogermacrene skeleton. This compound appears in six *Vitex* species and is one of the main products of *V. agnus-castus* [24,55,56,59,113] and *V. pseudo-negundo* [34,105,114]. OvTPS4 from oregano [115] and EgranTPS041 from *Eucalyptus* [116] were the first genes identified in plants responsible for the expression of a synthase that resulted in the production of bicyclogermacrene by heterologous expression. However, CmTPS1 from *Citrus medica* L. was the first gene responsible for the synthesis of bicyclogermacrene by homologous expression in vivo [117]. Although the gene responsible for the biosynthesis of bicyclogermacrene in *Vitex* species was not identified, its precursor was confirmed to be the germacradienyl cation (1.10-cyclization) in other plants (Figure 10) [47,118,119].

6,9-guaiadiene has a guaiane skeleton, which is rarely reported in plants, with two fused rings of five and seven carbons, respectively. It appears in five *Vitex* species, and it is the major compound of *V. gardneriana* [25,120]. δ-selinene synthase identified and characterized from *Abies grandis* catalyzed the formation of 34 different sesquiterpenes; among them, 6,9-guiadiene was one of the secondary products, with germacrene C as a precursor [121]. Although guaiane-type sesquiterpenes are common in nature and some enzymes described as producing guaianes as secondary reaction products were described [81,121], the guaiane synthases that catalyze the formation of this class of SS as their dominant reaction product were first reported in *Aquilaria crassna* [89]. Later, they were also found in *Aquilaria sinensis*, *Vitis vinifera*, and *Stellera chamaejasme* [122,123,124,125]. So far, *α* and *δ*-guaiene synthase were identified and characterized in these species with similar product profiles, with α or *δ*-guaiane as the main products and α-humulene and *β*-elemene in smaller amounts. In all these studies, germacrene A was the precursor of *α* or *δ*-guaiane.

**Figure 12:**
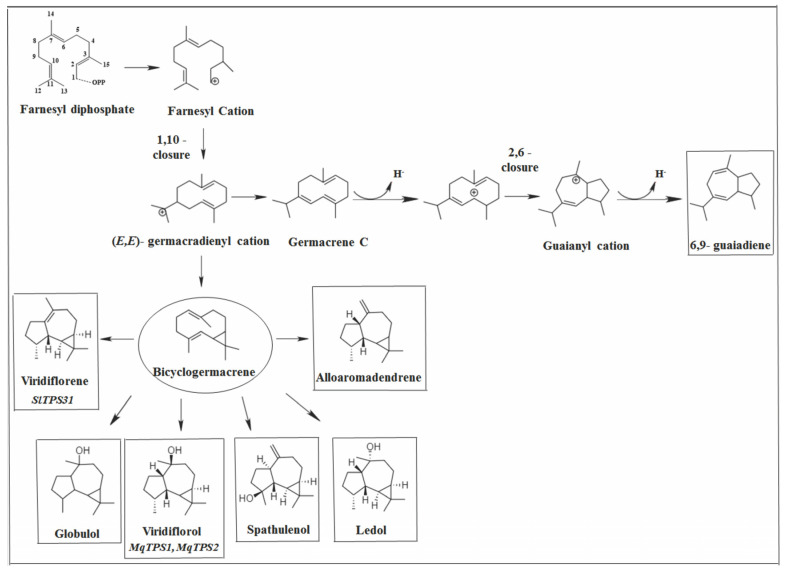
Types of primary cyclization of aromadrendanes, bicyclogermacrene, and 6,9-guiadiene.

It is postulated that 6,9-guaiadiene is synthesized through two cyclization reactions, the first constituting 1.10-cyclization to produce germacradienyl cation, which undergoes deprotonation in germacrene C. The second cyclization event occurs between C2 and C6 to generate the guaianyl carbocation followed by the subsequent deprotonation or addition of water (Figure 12) [89,122,125,126].

### 2.5. Tricyclic and Tetracyclic Sesquiterpenes

**Figure 13:**
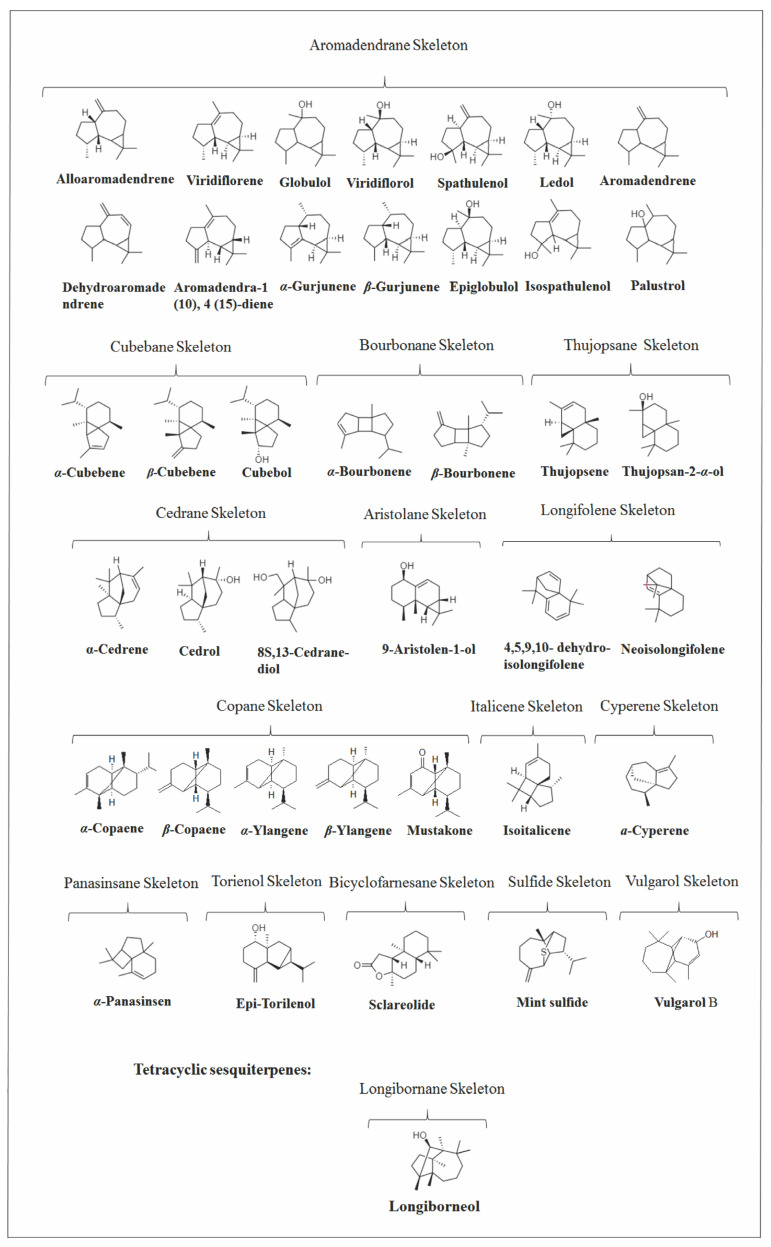
Structure of tricyclic and tetracyclic sesquiterpenes in *Vitex* species.

Thirty-nine tricyclic SS were identified in *Vitex* species (Figure 13). The aromadrendane skeleton was the most representative of this group with 14 compounds reported. It was the skeleton with the highest number of compounds within the criteria adopted in this work. Among them, allo-aromadendrane, spathulenol, globulol, viridiflorol, ledol, and viridiflorene were the most relevant. Allo-aromadrendene appeared in 10 species, with significant concentrations in *V. rufecens* [25] and *V. agnus-castus* [31,59]. Spathulenol was also identified in 10 species and is one of the main compounds of *V. agnus castus* [26,31], *V. rehmannii* [33], and *V. obovata* ssp. obovata (in lower concentrations) [33]. Globulol was reported in eight species and is the majority sequiterpene of the flowers of *V. negundo* [127] and the major SS in *V. zeyheri* [33]. Viridiflorol was identified in eight species and is the major compound in *V. negundo* [32,128]. It is also found in *V. agnus-castus* at lower concentrations [31]. Ledol was present in nine species, the secondary product being in *V. rufescens* [25]. Finally, viridiflorene was reported in seven species and was found in *V. capitata*, *V. megapotamica,* and *V. rufescens* in significant concentrations [25].

A small number of sesqui (TPS) specific for the formation of compounds from the aromadrendane skeleton in plants were identified. To date, *α*-gurjunene synthase from *Solidago canadensis* [129] and *Taiwania cryptomerioides* [130], viridiflorol synthase (MqTPS1 and MqTPS2) from *Melaleuca quinquenervia* [131], and viridiflorene synthase (SlTPS31) from *Solanum lycopersicum* [132] were reported in plant species. This is probably because the aromadrendane skeleton has the largest number of representative compounds in *Vitex* species, and specific synthases for the formation of these compounds may play an important role in the taxonomy of this genus.

The aromadendrane skeleton is characterized by the fusion of the gem-dimethylcyclopropane ring with the hydroazulane ring [133]. Several authors postulated that bicyclogermacrene is the biogenetic precursor of sesquiterpenoids with a gem-dimethylcyclopropane ring, including aromadendranes [95,133,134]. In addition, bicyclogermacrene is used as an intermediate platform for biomimetic access to various aromadendrane sesquiterpenoids, such as ledene, viridiflorol, palustrol, and spathulenol [135]. It was suggested that in *Psidium guineense* Sw., *Eucalyptus*, *Humulus lupulus*, and *Citrus junos* species, bicyclogermacrene is the key intermediate for aromadendrene derivatives [95,136,137]. However, in grapes and wines, the aromadendrane skeleton was reported to be structurally similar to the guaiane precursor. 6,11-cycloguaiane is referred to as an aromadendrane in which a cyclopropyl ring was formed by further cyclization of a guaiane precursor [46,47].

The catalysis of aromadrendanes in plants, the precursor being bicyclogermacrene or guaiane, as proposed in the literature, begins with 1.10-cyclization. This is supported by the previously proposed mechanism for the formation of viridiflorol based on quantum chemical calculations, starting with type 1.10 cyclization [84]. It was also proposed that the initial cyclization that originates viridiflorol in fungi is of the 1.10 type, although it occurs via the (*E*,*E*)–FPP and (3*R*)–NPP routes [138,139]. This indicates that viridiflorol biosynthesis in fungi can occur via both pathways.

The tetracyclic compound (Figure 13) identified was not representative within the criteria adopted in this review.

## 3. Insecticide and Response Activity of Sesquiterpenes Identified in *Vitex* Species

Plants are often exposed to attack by a variety of herbivorous arthropods and pathogenic microorganisms. In response to pest attacks, plants developed defense mechanisms to protect themselves [17,140]. Chemical defense strategies involve secondary metabolites, including SS, which can act directly through allelopathic or antimicrobial activity [27,140] or by indirect activation of systemic defenses in host and neighboring plants [17,141].

Sesquiterpenes are one of the main constituents of volatile mixtures released after damage by herbivorous insects or pathogens [140]. The induction of these compounds has frequently been reported as signaling molecules to attract natural enemies (predators and parasitoids) of herbivores, induce resistance responses against pathogens, and also act as precursors for the biosynthesis of sesquiterpenoid phytoalexins [13,17,111,140,142]. In addition, induced volatile mixtures can also play an important role in plant communication, functioning as airborne signals to induce defense in neighboring plants or to prepare unattacked plant tissue for defense responses to potential subsequent attack from herbivores [141,143,144].

Over the past two decades, studies showed evidence that sesqui (TPSs) and their corresponding products play a key role in defense in response to herbivory and phytopathogenic systems [140,145]. As an example, the induced rice sesqui (TPS) (OsSTPS2) gene plays a role in the antixenosis mechanism against the infestation of the brown gecko, *Nilaparvata lugens* [146]. Sesqui (TPS) from *Medicago truncatula* (MtTPS10) was specifically expressed in its roots after inoculation with the pathogen *Aphanomyces euteiches*, and its corresponding products inhibited mycelial growth and zoospore germination [145]. The longifolene synthase gene (PmTPS21) played a positive role in the defense mechanism of *Pinus massoniana* against the nematode, *Bursaphelenchus xylophilus* [147]. Two sesqui (TPS) (CsAFR and CsNSE2) from *Camellia sinensis* tea plants were up-regulated by damage from *Ectropis obliqua* Prout herbivores, emitting *α*-farnesene and (*E*)-nerolidol [148].

All aforementioned studies clearly showed the modulation of the plant defense against herbivores and pathogens through sesqui TPSs and their enzymatic terpenoid products. The following section summarizes the insecticidal activities and defensive responses of the main SS found in *Vitex* species.

### 3.1. Acyclic Sesquiterpenes

EβF is the main component of the aphid alarm pheromone, which is released by most aphid species when disturbed in the presence of predators and parasitoids [149,150]. This compound is detected in the bark oil of *Citrus junos* and in the leaves of the wild potato *Solatium berthaultii* Hawkes and is expected to play a similar role in these plants [62,151]. EβF can also induce oviposition in an aphidophagous float [152]. It can be used for biological control of aphids, releasing it in the field due to its deterrent and repellent effect in addition to attracting its natural enemies, such as predators and parasitic wasps (Hymenoptera: Braconidae) [153]. A previous study reported that inducible production of EβF via engineered TPS in genetically modified wheat may be necessary for the successful recruitment of natural enemies of the parasitic wasp *Aphidius ervi* [154]. Transgenic *Arabidopsis thaliana* produced large amounts of EβF, which showed a repellent effect for *Myzus persicae* [155].

Recently, a study found the expression of PvTPS16 and PvTPS02 genes in Switchgrass (*Panicum virgatum* L.) leaves, which are strongly correlated by the emission of high amounts of EβF, after treatment with the salicylic acid phytohormone, which simulates herbivory or infection by pathogens, and after treatment by *S. frugiperda* larvae [42]. The constitutive expression of the tps 46 gene reported in rice that is responsible for biosynthesis and constitutive emissions of Eβf may play a crucial role in the rice’s defense against *Rhopalosiphum padi* [156]. “It was suggested that constitutive release of defensive volatiles should occur when plants are growing in an environment where there is a high probability of herbivore attack” [156].

### 3.2. Monocyclic Sesquiterpenes

Recently, it was reported that *α*-humulene showed contact toxicity with high persistence after 48 h and repellency against the wheat grain pest *Sitophilus granarius* [157]. This compound was responsible, at least in part, for the deterrent effect of the oil of *Commiphora leptophloeos*, a spiny deciduous tree native to South America, causing deterrence from the oviposition of *A. aegypti* [158]. Furthermore, *α*-humulene showed strong contact activity against the cigarette beetle (*Lasioderma serricorne*) and was one of the components of the essential oil of *Piper aduncum* responsible for repelling the *Tetranychus urticae* mite [159,160]. After treatment with methyl jasmonate (MeJa), an elicitor of plant defensive responses, the AcHS1–3 gene up-regulated *α*-humulene synthase expression in *Aquilaria crassina* cell culture [75].

Germacrene D was implicated in plant-insect interactions. It is used to select host plants by the antenna receptors of the caterpillar tobacco moth *Heliothis virescens* [161]. It can also act as an anti-attractant to protect plants from beetle attacks [162]. They are repellent to aphids and bovine ticks [154,163,164]. Tozin et al. [165] identified a 126% increase in germacrene D in glandular trichomes of *Ocimum gratissimum* after attacks by leaf-cutting ants, *Acromyrmex rugosuse*.

Elemenes are natural sesquiterpenes present in essential oils in a mixture of *β*-elemene, *γ*-elemene, and *δ*-elemene. *β*-elemene showed significant toxic effects on fall armyworm *Spodoptera exigua* (Hubner) [166]. Taniguchi et al. [86] identified that the *β*-elemene synthase gene in rice was up-regulated by treatment with the plant hormone jasmonic acid (JA), which works as a signaling molecule in the regulation of plant defense. In the same study, it was reported to have antifungal activity against the rice pathogen *Magnaporthe oryzae*.

### 3.3. Bicyclic Sesquiterpenes

Cadinene is a group of sesquiterpenes with isomeric hydrocarbons, including *δ*-cadinene, that were implicated in the defense of the cotton plant against pathogens and pests [40,167]. Several *δ*-cadinene synthases were already identified and characterized in cotton species and are responsible for producing *δ*-cadinene, the precursor for the biosynthesis of cadinane-type phytoalexins, such as gossypol [40,142]. This is an important arthropod resistance compound that provides constitutive and inducible defense against cotton pests and diseases [167,168]. The expression of the *δ*-cadinene synthase gene was induced by rhizosphere bacteria, and plants that produced *δ*-cadinene were considered resistant to *Spodoptera exigua* (Hubner) [168]. Oxidative cadinene showed significant antifungal and antibacterial activities against phytopathogenic fungi and bacteria [169,170].

EβC is involved in the indirect defense of several plants, attracting the natural enemies of above and below-ground pests [12,17,171,172]. The attack of herbivorous insects or treatment with MeJa induced the expression of genes responsible for the transcription of EβC synthase from corn (ZmTPS23), rice (OsTPS3), sorghum (SbTPS4), cotton (GhTPS1), and Switchgrass (PvTPS14), which were responsible for the emission of EβC, attracting herbivore parasitoids and entomopathogenic nematodes [16,42,172,173,174,175]. In addition, EβC can also act in direct defense against bacterial pathogens that invade floral tissues [27]. A previous study showed that caryophyllene-rich rhizome oil from *Zingiber nimmonii* has a significant inhibitory activity against *Bacillus subtilis* and *Pseudomonas aeruginosa* bacteria [176]. Previous studies also reported that EβC and caryophyllene oxide decreased the growth and survival of *Heliothis virescens* and *Hymenaea species* [177,178].

Caryophyllene oxide showed toxicity against the aphid *Metopolophium dirhodum* (Hemiptera: Aphididae), and in mixtures with citral and EβC, it was also effective against the aphid *Myzus persicae* [108,179]. This compound also showed excellent repellent properties against *A. aegypti* and *Anopheles minimus* mosquitoes, with better performance than the commercial repellent N,N-diethyl-meta-toluamide (DEET) [109]. Furthermore, it is one of the main constituents of the oil of *Artabotrys hexapetalus* Bhandari, which was shown to have strong repellent activity against females of *Anopheles gambiae*, a species of malaria vector in Africa [180].

Although sesquiterpenes belonging to the selinene family were widely reported in different plants, there are limited studies investigating the insecticidal activity of *β*-selinene. However, this compound was detected in corn only in the context of pathogen attack [181,182]. Ding et al. [111] reported *β*-selinene synthase (ZmTps21) in maize being transcribed after fungal elicitation, long-term root herbivory, and combined field pressures. Its products *β*-selinene and its nonvolatile acid derivative, *β*-costic acid inhibited the growth of pathogenic fungi and corn root larvae (*Diabrotica balteata*). A previous study identified the presence of ZmTps21 in the transcriptome analysis of resistant maize lines associated with enhanced antifungal defenses [183]. It was suggested that *α*-selinene from TPS05 in switchgrass roots serves as a precursor of *α*-costic acid, which may exhibit similar functions in the antimicrobial defense of this plant. *β*-selinene also showed contact toxicity against the vinegar fly, *Drosophila melanogaster* [184].

There are no reports that bicyclogermacrene, as a nonoxygenated sesquiterpene, has insecticidal activity; however, its non-volatile oxygenated derivatives, such as Mandolin A and Parteniol, showed an inhibitory activity on acetylcholinesterase and fungistatic activity against the growth of *Aspergillus niger* [133,185,186].

6,9-guaiadiene was the major compound of the essential oil of *V. gardneriana*, which showed acaricide and larvicide activity against *Aceria guerreronis* and *A. aegypty*, respectively [25,120]. Studies showed that the gene expression in *Aquilaria* species was up-regulated, encoding *δ*-guiene synthase in response to mechanical injury and MeJa treatment and inducing *δ*-guaiene production [126,187,188]. Recently, transcriptome analysis of western aspen-balsam infected roots (*Populus trichocarpa*) by *Phytophthora cactorum* (Oomycetes) revealed the induction of the PtTPS5 gene, forming the compounds (1S, 5S, 7R, 10R)-guaia-4(15)-en-11-ol and (1S, 7R, 10R)-guaia-4-en-11-ol [189].

### 3.4. Tricyclic Sesquiterpenes

In this group of sesquiterpenes, some aromadrendane compounds showed insecticidal activity due to the conformational rigidity that the gem-dimethylcyclopropyl group imposes, the lipophilic character of the methyl groups, and the variation in oxygen functions between the compounds; it can favor the binding with lipoprotein receptors, triggering several biological responses, including insecticidal activity [133]. The compound spathulenol, for example, showed toxicity against the aphid *Metopolophium dirhodum* (Hemiptera: Aphididae) and two types of insects from stored products, *Tribolium castaneum* and *Lasioderma serricorne* [190,191]. This compound also showed repellency against mosquitoes (*A. stephensi* and *A. aegypti*), a leaf-cutting ant (*Atta cephalotes*), a red flour beetle (*Tribolium castaneum*), and a smoke beetle (*Lasioderma serricorne*) [191,192,193]. Furthermore, antifungal activity against the pathogen affecting cucumber crops, *Cladosporium cucumerinum*, was reported [194]. Allo-aromadendrane and its derivative, alloaromadendrane-4β,10β-diol, were effective inhibitors of the growth of the fungi *Cladosporium herbarum* and *P. oryzae* [195,196].

The compound viridiflorol also showed antifungal activity, inhibiting the growth of phytopathogenic fungi, *Colletotrichum truncatum*, *Pyricularia oryzae*, and *Cladosporium cucumerinum* [138,194,197]. A diet rich in this compound was able to reduce the fecundity and survival of melaleuca weevil larvae (*Oxyops vitiosa*) and influence the oviposition of *Boreioglycaspis melaleucae* adults [198,199]. Like the compounds mentioned above, globulol also showed activity against the phytopathogenic fungus *C. cucumkrinum* [194]. Furthermore, it was emitted in larger quantities in *Eucalyptus benthamii* after the herbivory of the bronze insect, *Thaumastocoris peregrinus*, indicating that this compound is involved in defensive strategies of this plant [200].

## 4. Discussion

The diversity of sesquiterpenes in *Vitex* species draws attention to a possible significant expression of genes encoding sesquiterpene synthases. The most relevant and representative sesquiterpenes of the genus *Vitex* mentioned in this review are derived from the germacredienyl cation, including the bicyclogermacrene pathway, which gives rise to aromadrendanes as the largest number of representative compounds in the genus, and the germacrene C pathway, which forms the rare compound 6,9-guiadiene in plants. This indicates that 1.10-cyclizing sesquiterpene synthases responsible for the formation of these compounds may play an important role in the taxonomy of the genus and in the chemosystematics among species. A previous study by our research group that used a metabolomic approach, molecular markers, and statistical analysis through a clustering algorithm identified a notable presence of aromadrendane compounds in four plants collected in northeastern Brazil, suggesting that aromadrendanes ring closure can be considered a more specific signature of the chemical profile for species in the *Vitex* genus [25].

Much was discussed in recent decades about the great taxonomic redelimitation of Lamiaceae and Verbenaceae. This was confirmed by [201] using morphological markers and later consolidated by [202] using molecular markers from conserved parts of chloropaste of different species distributed in several subfamilies. As a result, an important part of the Verbenaceae family was redistributed among several subfamilies in Lamiaceae, including Viticoideae, which contains *Vitex* as the largest genus. However, Viticoideae was recognized as the least satisfactory among the subfamilies that were circumscribed, with morphological, phytochemical, and molecular evidence suggesting it as clearly paraphyletic and possibly polyphyletic [201]. In the phylogenetic study by [202], Neptododeae belongs to a clade very close to Vitcoideae, evidencing a genetic proximity between these subfamilies. Interestingly, aromadrendadanes were proposed as chemotaxonomic markers for the genera *Marsypianthes* and *Hypenia*, which belong to Neptododeae [203,204]. Therefore, it is suggested that sesquiterpene synthases and their cyclization mechanism for the formation of aromadrendanes may be correlated with this proximity of the clades, indicating a conserved base of genes among these subfamilies, constituting an interesting approach that can help in the development of a better understanding of the taxonomy of the family Lamiaceae.

In addition to 1.10-cyclization, sesquiterpenoids derived from 1.6-cyclization as well as a 1.11-cyclization mechanism were also identified in *Vitex*. These enzymes were found to appear to group together not only according to gene sequence similarity but also by cyclization mechanism [205]. Phylogenetic analysis in fungi allowed us to offer a predictive framework for the targeted discovery of new sesquiterpene synthases based on the cyclization mechanism of choice, streamlining the identification and cloning of new sesquiterpene synthases that produce desirable natural products [205,206]. The availability of an increasing number of sesquiterpene synthases characterized in plants opens the door to the application of computational predictive phylogenetic analysis to obtain information about this surprisingly diverse family of enzymes. This may contribute to a greater understanding of how this gene family is organized and how it has evolved over time. Additionally, by deepening our understanding of carbocation chemistry from the cyclization products of these enzymes, we can also develop tools for the biosynthetic production of relevant insecticidal compounds that may not be accessible by traditional chemical syntheses.

## 5. Conclusions

This review considers the strong presence of sesquiterpenes in *Vitex* species. The pathways and mechanisms proposed for the biosynthesis of identified sesquiterpenes were broadly summarized based on data found in the literature. This provides new insights for a deeper understanding of taxonomy information about the biosynthesis of sesquiterpenes in this genus through gene expression. Data and information on the expression for the formation of enzymes responsible for the biosynthesis of sesquiterpenes in *Vitex* plants are scarce and require further investigation.

Modulation of plant defense against herbivores and pathogens through sesqui (TPSs) and their terpenoid enzymatic products indicate the importance and value of plants that are rich in sesquiterpenes. For a comprehensive understanding of sesquiterpenes in *Vitex* species, further studies should focus on confirming their biosynthesis pathway and the influence of herbivores and pathogens on the gene regulation and expression mechanism, elucidating their importance in the defense process of the *Vitex* plant.

## Data Availability

The data presented in this article are available on request from the corresponding author.
